# Native *Trichoderma* Induced the Defense-Related Enzymes and Genes in Rice against *Xanthomonas oryzae* pv. *oryzae* (*Xoo*)

**DOI:** 10.3390/plants12091864

**Published:** 2023-04-30

**Authors:** Md. Rashidul Islam, Rabin Chowdhury, Arpita Saha Roy, Md. Nazmul Islam, Mamuna Mahjabin Mita, Samrin Bashar, Plabon Saha, Ridwan Ahmed Rahat, Mehedi Hasan, Mst. Arjina Akter, Md. Zahangir Alam, Md. Abdul Latif

**Affiliations:** 1 Plant Bacteriology and Biotechnology Laboratory, Department of Plant Pathology, Bangladesh Agricultural University, Mymensingh 2202, Bangladesh; 2 Faculty of Agriculture, Bangladesh Agricultural University, Mymensingh 2202, Bangladesh; 3 Plant Pathology Division, Bangladesh Rice Research Institute, Joydebpur, Gazipur 1701, Bangladesh

**Keywords:** bacterial blight, antimicrobial activity, resistance, defense signaling

## Abstract

The application of *Trichoderma* is a form of biological control that has been effective in combating *Xanthomonas oryzae* pv. *oryzae*, the causative agent of the devastating disease known as bacterial blight of rice. In this present study, four strains of *Trichoderma,* viz., *T. paraviridescens* (BDISOF67)*, T. erinaceum* (BDISOF91), *T. asperellum* (BDISOF08), and *T. asperellum* (BDISOF09), were collected from the rice rhizosphere and used to test their potentiality in reducing bacterial blight. The expression patterns of several core defense-related enzymes and genes related to SA and JA pathways were studied to explore the mechanism of induced resistance by those *Trichoderma* strains. The results primarily indicated that all *Trichoderma* were significantly efficient in reducing the lesion length of the leaf over rice check variety (IR24) through enhancing the expression of core defense-related enzymes, such as PAL, PPO, CAT, and POD activities by 4.27, 1.77, 3.53, and 1.57-fold, respectively, over control. Moreover, the results of qRT-PCR exhibited an upregulation of genes *OsPR1*, *OsPR10*, *OsWRKY45*, *OsWRKY62*, *OsWRKY71*, *OsHI-LOX*, and *OsACS2* after 24 h of inoculation with all tested *Trichoderma* strains. However, in the case of RT-PCR, no major changes in *OsPR1* and *OsPR10* expression were observed in plants treated with different *Trichoderma* strains during different courses of time. Collectively, *Trichoderma* induced resistance in rice against *X. oryzae* pv. *oryzae* by triggering these core defense-related enzymes and genes associated with SA and JA pathways.

## 1. Introduction

Rice (*Oryzae sativa* L.) is one of the major cereal crops belonging to the Poaceae family, especially in Asian countries including China, India, Bangladesh, Thailand, and Vietnam [1,2]. With a constantly rising production of 3.6 crore tons of rice, Bangladesh stands in the third position globally in rice production after China and India [3]. However, bacterial blight (BB) caused by *Xanthomonas oryzae* pv. *oryzae* (Xoo) is among the thirty-two rice diseases reported in Bangladesh, which can cause 5.8–30.4% yield loss depending on the crop stage and environmental factors [4,5]. For systemic bacterial blight infection, *X. oryzae* pv. *oryzae* enters the rice leaf generally by wounds or water pores of wavy leaf edges, multiplies in the intercellular spaces of the underlying epithelial tissues, and then moves to the xylem vessels [6].“Within a few days bacterial cells and EPS fill the xylem vessels and ooze out from hydathodes, forming beads or strands of exudate on the leaf surface, a characteristic sign of the disease and a source of secondary inoculum” [7].

Chemical management, biological control, and genetic resistance have all been used to manage bacterial leaf blight disease in rice plants. Despite the fact that the rising population encourages the use of more chemical inputs, such management strategies are not rewarding due to their adverse effect on the consumers, ecosystem, land productivity, and the variable susceptibility of different pathogenic races to the chemicals used to control them [8,9,10]. This study aimed to develop environment-friendly sustainable control measures against the bacterial blight of rice. A good number of *Trichoderma* strains have been found to be effective as biocontrol agents against *X. oryzae* pv*. oryzae* under greenhouse and field conditions [11,12,13]. Recently, Mishra et al. have shown that *T. atroviride* induces rice plant growth and suppresses the in vitro growth of *X. oryzae* pv*. oryzae* [14]. The mechanism by which *Trichoderma* induces resistance against biotic and abiotic stresses is through the direct release of certain chemical compounds, antibiotics, lytic enzymes, and hormones, or indirectly by competing for a niche or nutrients and inducing systemic resistance (ISR) [11,15,16,17]. Studies have shown that talc-based powder formulations of *Trichoderma* were used for field evaluation and showed a significant result in controlling BB of rice [18,19].

The pathogenicity of *X. oryzae* pv. *oryzae* largely depends on the coordinated expression of host-induced genes/proteins, including systemic signals such as salicylic acid (SA) and jasmonic acid (JA) in vivo and in vitro [20,21]. Recent studies showed that *Trichoderma* sp. induces systemic acquired resistance (SAR) through SA, and induced systemic resistance (ISR) through JA and ethylene (ET) signal pathways, which are associated with the activation of plant defense mechanisms [22,23,24,25,26]. These mechanisms include changes in biochemistry [27], activation of multiple defense-related enzymes [28], and induction of pathogenesis-related (PR) proteins [29]. Peroxidase (POD) is one of the fast-responding defense-related enzymes against plant pathogens which are precisely involved in lignification, suberification, polymerization of hydroxyproline-rich glycoproteins, regulation of cell wall elongation, wound healing, and resistance against pathogens in plants [28,30]. Catalase (CAT) is an oxygen-scavenging enzyme that protects cells from the toxic effects of H_2_O_2_ during development by converting it to water and molecular oxygen [31,32]. Polyphenol oxidase (PPO) is an enzyme in plants that regulates feeding and growth, and plays a major role in plant defense against biotic and abiotic stresses [33]. Phenylalanine ammonia-lyase (PAL) appears to be one of the vital elements of ISR, the biosynthesis of SA, an essential signal involved in induced systemic resistance [34], and plays a significant role in the synthesis of several defense-related secondary compounds such as phenols and lignin [35,36]. Therefore, *Trichoderma* can induce plant resistance through ISR, which further increases the activity of the POD, PPO, CAT, and PAL enzymes in rice leaves [37,38,39,40,41].

An important characteristic of induced resistance is the phenomenon of priming, in which rice plants exhibit a more rapid and elevated expression of defense responses upon bacterial pathogen infection compared to untreated rice plants [42]. As part of induced resistance, systemic signals such as SA and JA influence the pathogen’s virulence machinery. Studies reported that the *OsPR1*, *OsPR10*, *OsWRKY45*, *OsWRKY62*, *OsWRKY71*, *OsHI-LOX*, and *OsACS2* genes relevantly upregulated in rice plants were found to develop resistance to *X. oryzae* pv. *oryzae* [20,41,43,44,45,46,47,48,49]. However, *OsPR1* and *OsPR10* are well-known pathogenesis-related genes of rice that are induced by high SA levels [50,51]. In addition, the rice genome contains more than 100 WRKY genes, and OsWRKY45, *OsWRKY62*, and OsWRKY71 play important roles in the monitoring of genes involved in pathogen-induced defense responses [52,53,54,55,56]. BB resistance tests also showed that *OsACS2* (1-aminocyclopropane-1-carboxylic acid synthase) is a key enzyme of ET biosynthesis under the control of a strong pathogen-inducible promoter. *Trichoderma* activates the octadecanoic pathway in plant roots with *Lox1* being activated immediately, which apparently leads to the production of JA signals [38]. However, this present study was carried out to evaluate the potentiality of *Trichoderma* isolates in controlling BB of rice. Moreover, we assessed the expression patterns of several core defense-related enzymes and genes which are related to SA and JA pathways to explore the mechanism of induced systemic resistance by *Trichoderma* strains in controlling *X. oryzae* pv. *oryzae*.

## 2. Results

### 2.1. Antagonistic Activity of Some Fungal Isolates against X. oryzae pv. oryzae

**Figure 1:**
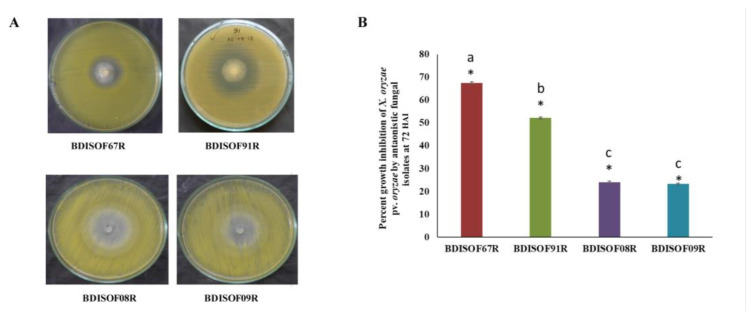
(**A**) In vitro growth inhibition of *X. oryzae* pv. *oryzae* by antagonistic fungal isolates (BDISOF67R: obtained from rice rhizosphere from Bogura; BDISOF91R: obtained from rice rhizosphere from Hobigonj; BDISOF08R and BDISOF09R: both obtained from rice rhizosphere from Jashore) (Photographs taken after 72 h of inoculation). (**B**)**.** Error bar chart of percent growth inhibition of *X. oryzae* pv. *oryzae* by antagonistic fungal isolates. (* represents significance at 5% level of significance. Similar letters at the top of bars indicate the treatment means are statistically similar).

A total of 200 fungal isolates were isolated and purified from the rice rhizosphere. Out of these, four fungal isolates, viz., BDISOF67R, BDISOF91R, BDISOF08R, and BDISOF09R, were identified as antagonists against *X. oryzae* pv. *oryzae* (Figure 1A) that inhibited the growth of *X. oryzae pv. oryzae* by 67.51%, 52.19%, 24.06%, and 23.33%, respectively, over control (Figure 1B).

### 2.2. Molecular Identification of Fungal Isolates BDISOF67R, BDISOF91R, BDISOF08R, and BDISOF09R

**Figure 2:**
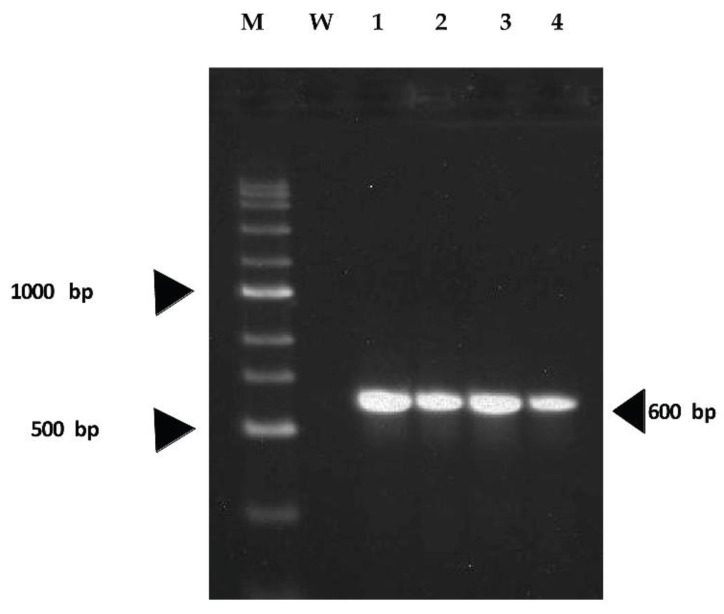
PCR confirmation by amplification of the ITS region of the beneficial fungi identified from rhizosphere samples collected in Boro season 2019 that inhibit the growth of *X. oryzae* pv. *oryzae*. (M: DNA ladder; W: Water control; 1: *T. paraviridescens* (BDISOF67); 2: *T. erinaceum* (BDISOF91); 3: *T. asperellum* (BDISOF08); 4: *T. asperellum* (BDISOF09)).

Fungal isolates BDISOF67R, BDISOF91R, BDISOF08R, and BDISOF09R were identified by PCR amplification of the internal transcribed spacer (ITS) region using ITS-1 and ITS-4 primers and PCR products were then sequenced. The results of PCR showed an amplification size of 600 bp and confirmed that the antagonist organisms are fungi (Figure 2).

Analysis of sequencing data of the amplified ITS region using BLAST program revealed that BDISOF67R, BDISOF91R, BDISOF08R, and BDISOF09R showed the highest homology with *Trichoderma paraviridescens* strain 36114DRJ (accession #MF782827.1), *Trichoderma erinaceum* strain QT22079 (accession #KY225644.1), *Trichoderma asperellum* bio-material USM (accession #KU878976.1), and *Trichoderma asperellum* isolate 20B (accession #MZ044276.1), which confirmed that the fungal species are *Trichoderma paraviridescens* (BDISOF67R) (accession # OP456159), *Trichoderma erinaceum* (BDISOF91R) (accession # OP456160), *Trichoderma asperellum* (BDISOF08R) (accession # OP456157), and *Trichoderma asperellum* (BDISOF09R) (accession # OP456158), respectively (Table 1).

### 2.3. Effect of Some Selected Antagonistic Trichoderma Isolates on the Reduction of Lesion Length in Susceptible Check Cultivar (IR24) Caused by X. oryzae pv. oryzae

**Figure 3:**
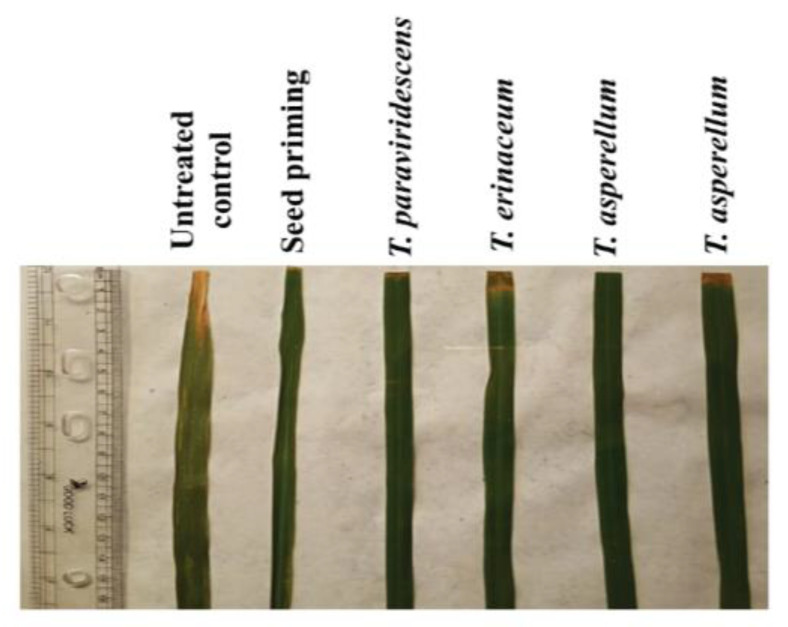
Photograph showing the reduction of lesion length caused by *X. oryzae* pv. *oryzae* by *Trichoderma* isolates in susceptible check cultivar (IR24). Photographs were taken at 14 days after inoculation with *X. oryzae* pv. *oryzae* by leaf clipping method. Untreated control: seed treatment with only distilled water.

The results of plant inoculation (rice seeds first treated with *Trichoderma* formulations and then foliar application of *Trichoderma* spp.) showed a significant reduction of lesion length in plants sprayed with formulated *Trichoderma* spp. as compared with untreated control (Figure 3; Table 2). 

The minimum (3.83 mm) lesion length was observed in plants sprayed with BDISOF08 *(T. asperellum),* followed by BDISOF67 (*T. paraviridescens)* (5.50 mm), BDISOF91 (*T. erinaceum)* (10.83 mm), and BDISOF09 (*T. asperellum)* (15.50 mm), while the maximum (87%) reduction of lesion length was observed in plants sprayed with BDISOF08 *(T. asperellum),* followed by BDISOB45R (*T. paraviridescens)* (75.00%), and BDISOF91 (*T. erinaceum)* (60.42%). The minimum (31.25%) reduction of lesion length was observed in plants sprayed with BDISOF09 (*T. asperellum)*. 

### 2.4. Differential Expression of Some Defense-Related Enzymes in Rice Induced by Selected Trichoderma Isolates in Response to X. oryzae pv. oryzae

#### 2.4.1. Effect of Selected Trichoderma Isolates on Phenylalanine Ammonia-Lyase (PAL) Activity in Response to *X. oryzae* pv. *oryzae*

As Table 3 shows, plants treated with BDISOF67 (*T. paraviridescens*) showed the maximum (609 μmol transcinnamic acid min^−1^ g^−1^ protein) PAL activity at 48 HAI, while the minimum (527 μmol transcinnamic acid min^−1^ g^−1^ protein) PAL activity was recorded at 72 HAI, which were 2.55 and 3.33 times higher over control, respectively.

On the other hand, the maximum (647 μmol transcinnamic acid min^−1^ g^−1^ protein) PAL activity was recorded for plants treated with BDISOF91 (*T. erinaceum*) at 72 HAI, while the minimum (555 μmol transcinnamic acid min^−1^ g^−1^ protein) PAL activity was at 48 HAI, which showed 4.05 and 2.32 times more PAL activity over control, respectively. However, plants treated with BDISOF08 (*T. asperellum*) showed the maximum (706 μmol transcinnamic acid min^−1^ g^−1^ protein) PAL activity at 24 HAI, while the minimum (530 μmol transcinnamic acid min^−1^ g^−1^ protein) PAL activity was recorded at 72 HAI, which were 2.85 and 3.32 times higher over control, respectively. Lastly, plants treated with BDISOF09 (*T. asperellum*) accounted for the maximum (682 μmol transcinnamic acid min^−1^ g^−1^ protein) PAL activity at 72 HAI, while the minimum (556 μmol transcinnamic acid min^−1^ g^−1^ protein) PAL activity was recorded at 24 HAI, which demonstrated 4.27 and 2.25 times higher PAL activity over control, respectively.

#### 2.4.2. Influence of Selected Trichoderma Isolates in Induction of Catalase (CAT) Activity in Response to *X. oryzae* pv. *oryzae*

From Table 4, plants treated with BDISOF67 (*T. paraviridescens*) showed the maximum (273 µmol H_2_O_2_ min^−1^ g^−1^) CAT activity at 24 HAI, while the minimum (162 µmol H_2_O_2_ min^−1^ g^−1^) CAT activity was recorded at 72 HAI, which were 5.46 and 1.34 times higher over control, respectively.

On the other hand, the maximum (264 µmol H_2_O_2_ min^−1^ g^−1^) CAT activity was recorded for plants treated with BDISOF91 (*T. erinaceum*) at 24 HAI, while the minimum (118 µmol H_2_O_2_ min^−1^ g^−^1) CAT activity was at 48 HAI, which showed 5.28 and 0.98 times more CAT activity over control, respectively. However, plants treated with BDISOF08 (*T. asperellum*) showed the maximum (201 µmol H_2_O_2_ min^−1^ g^−1^) CAT activity at 144 HAI, while the minimum (59 µmol H_2_O_2_ min^−1^ g^−1^) CAT activity was recorded at 72 HAI, which were 1.94 and 0.49 times higher over control, respectively. Lastly, plants treated with BDISOF09 (*T. asperellum*) accounted for the maximum (367 µmol H_2_O_2_ min^−1^ g^−1^) CAT activity at 144 HAI, while the minimum (78 µmol H_2_O_2_ min^−1^ g^−1^) CAT activity was recorded at 72 HAI, which demonstrated 3.53 and 0.64 times higher CAT activity over control, respectively. 

#### 2.4.3. Effect of Selected Trichoderma Isolates in Induction of Polyphenoloxidase (PPO) Activity in Response to *X. oryzae* pv. *oryzae*

As is illustrated by Table 5, plants treated with BDISOF67 (*T. paraviridescens*) showed the maximum (454 units g^−1^ min^−1^ FW) PPO activity at 24 HAI, while the minimum (247 units g^−1^ min^−1^ FW) PPO activity was recorded at 72 HAI, which were 1.77 and 1.55 times higher over control, respectively.

On the other hand, the maximum (434 units g^−1^ min^−1^ FW) PPO activity was recorded for plants treated with BDISOF91 (*T. erinaceum*) at 24 HAI, while the minimum (271 units g^−1^ min^−1^ FW) PPO activity was at 72 HAI, which showed 0.95 and 1.7 times more PPO activity over control, respectively. However, plants treated with BDISOF08 (*T. asperellum*) showed the maximum (428 units g^−1^ min^−1^ FW) PPO activity at 24 HAI, while the minimum (222 units g^−1^ min^−1^ FW) PPO activity was recorded at 144 HAI, which were 0.98 and 0.82 times higher over control, respectively. Lastly, plants treated with BDISOF09 (*T. asperellum*) accounted for the maximum (403 units g^−1^ min^−1^ FW) PPO activity at 24 HAI, while the minimum (284 units g^−1^ min^−1^ FW) PPO activity was recorded at 48 HAI, which demonstrated 0.94 and 1.19 times higher PPO activity over control, respectively.

#### 2.4.4. Influence of Selected Trichoderma Isolates in Induction of Peroxidase (POD) Activity in Response to *X. oryzae* pv. *oryzae*

Table 6 indicates that plants treated with BDISOF67 (*T. paraviridescens*) showed the maximum (1751 units min^−1^ g^−1^ FW) POD activity at 144 HAI, while the minimum (850 units min^−1^ g^−1^ FW) POD activity was recorded at 24 HAI, which were 1.47 and 1.12 times higher over control, respectively.

On the other hand, the maximum (1866 units min^−1^ g^−1^ FW) POD activity was recorded for plants treated with BDISOF91 (*T. erinaceum*) at 144 HAI, while the minimum (831 units min^−1^ g^−1^ FW) POD activity was at 24 HAI, which showed 1.57 and 1.09 times more POD activity over control, respectively. However, plants treated with BDISOF08 (*T. asperellum*) showed the maximum (1809 units min^−1^ g^−1^ FW) POD activity at 144 HAI, while the minimum (697 units min^−1^ g^−1^ FW) POD activity was recorded at 72 HAI, which were 1.52 and 0.69 times higher over control, respectively. Lastly, plants treated with BDISOF09 (*T. asperellum*) accounted for the maximum (1704 units min^−1^ g^−1^ FW) POD activity at 72 HAI, while the minimum (762 units min^−1^ g^−1^ FW) POD activity was recorded at 48 HAI, which demonstrated 1.69 and 1.06 times higher POD activity over control, respectively.

### 2.5. Differential Expression of Some SA and JA Pathway Related Genes in Plants Treated with Trichoderma 

#### 2.5.1. Expression Levels of Some Selected Defense Related Genes Involved in Salicylic (SA) and Jasmonic (JA) Acid Pathways by RT-PCR

**Figure 4:**
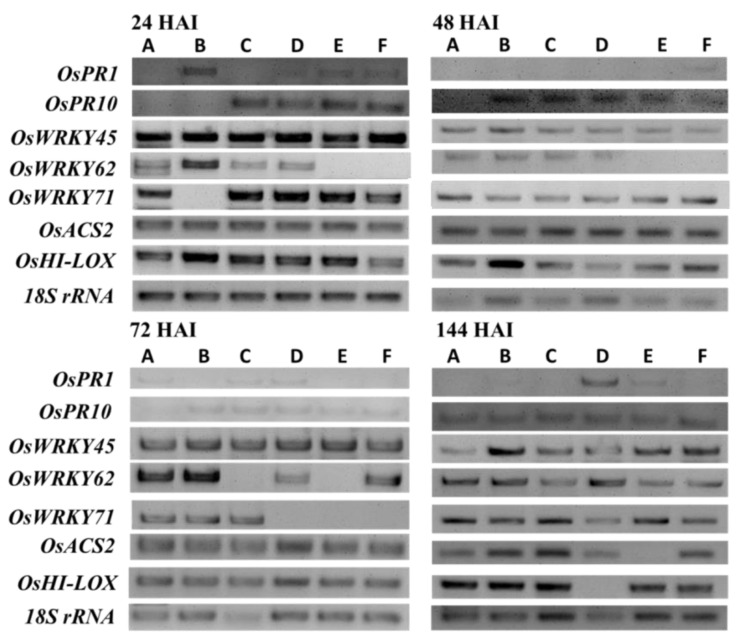
Expression levels of some selected marker genes involved in SA and JA acid pathways by RT-PCR. Total RNA was extracted from rice leaves and cDNA was synthesized. PCR was performed using cDNA as template. (A) untreated control; (B) seed priming; (C) *T. paraviridescens* (BDISOF67); (D) *T. erinaceum* (BDISOF91); (E) *T. asperellum* (BDISOF08); (F) *T. asperellum* (BDISOF09). Original images for RT-PCR image are provided in Supplementary Figures S1–S4.

No major changes in *OsPR1* and *OsPR10* expression were observed in plants treated with different *Trichoderma* strains as compared with untreated and positive control at 144 HAI (Figure 4).

However, when BDISOF67 (*T. paraviridescens*), BDISOF91 (*T. erinaceum*), BDISOF08 (*T. asperellum*), and BDISOF09 (*T. asperellum*) were sprayed on plants, levels of *OsWRKY45, OsWRKY62,* and *OsWRKY71* expression were found to be elevated at 144 HAI as compared with untreated and positive control (Figure 4). Furthermore, there was an increase in *OsACS2* and *OsHI-LOX* expression in plants sprayed with *T. paraviridescens* (BDISOF67) and *T. asperellum* (BDISOF09) at 144 HAI (Figure 4). However, plants treated with *T. erinaceum* (BDISOF91) did not show any expression of *OsHI-LOX* genes at 144 HAI, but were found to be elevated in the case of *OsACS2* genes at 144 HAI as compared with untreated control (Figure 4). On the other hand, *OsHI-LOX* expression level was found to be elevated at 144 HAI when plants were treated with *T. asperellum* (BDISOF08) as compared to untreated control, but did not get expressed in the case of *OsACS2* genes at the same period (Figure 4). To summarize, these results primarily indicated that *Trichoderma* spp. reduced bacterial blight severity in rice by inducing the expression of some selected SA and JA pathway-related genes (Figure 4). 

#### 2.5.2. Expression Levels of Some Selected Defense-Related Genes Involved in Salicylic (SA) and Jasmonic (JA) Acid Pathways by Real-Time PCR (q-PCR)

**Figure 5:**
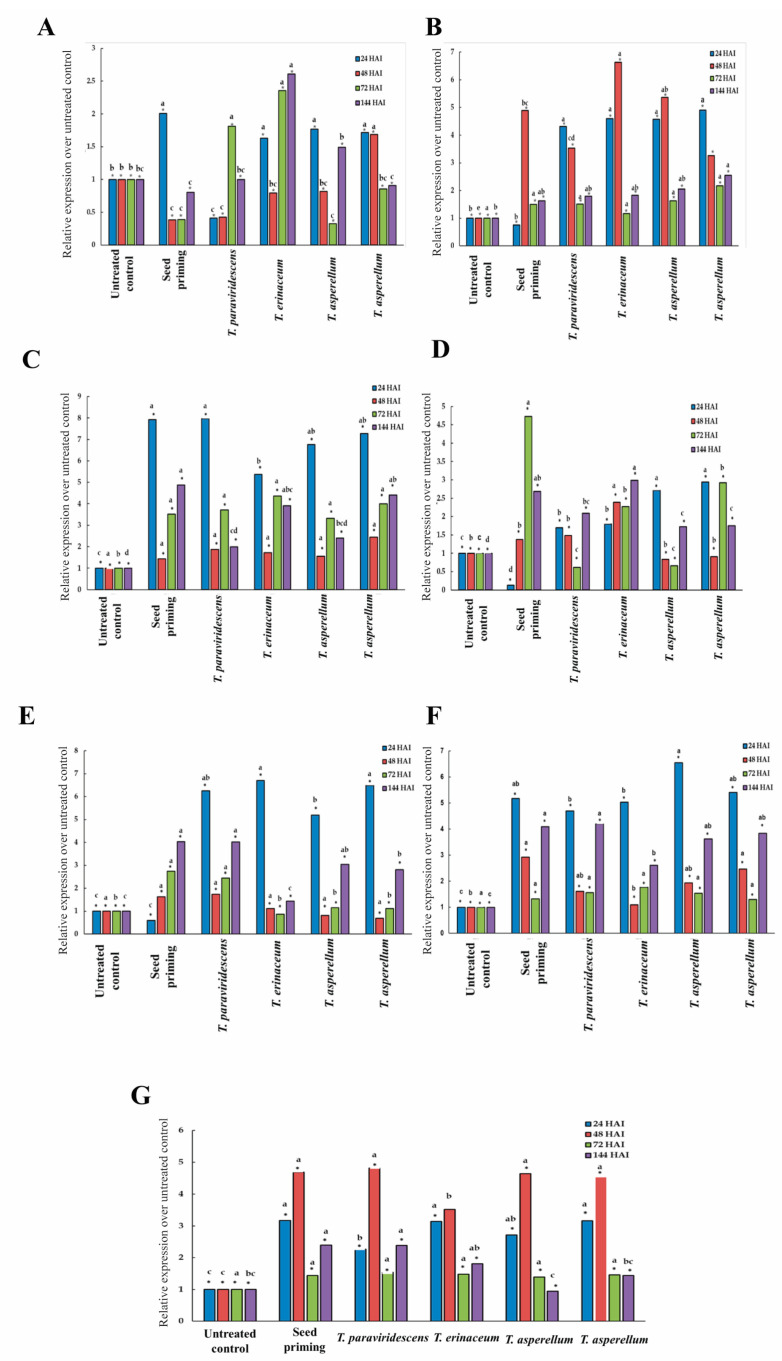
Expression levels of (**A**) *OsPR1*, (**B**) *OsPR10,* (**C**) *OsWRKY45,* (**D**) *OsWRKY62*, (**E**) *OsWRKY71*, (**F**) *OsHI-LOX,* and (**G**) *OsACS2* involved in SA and JA acid pathways by q-PCR in rice treated with *Trichoderma* in response to *X. oryzae* pv*. oryzae* over untreated control. Untreated control: seed treatment with only distilled water (*T. paraviridescens* (BDISOF67), *T. erinaceum* (BDISOF91), *T. asperellum* (BDISOF08), and *T. asperellum* (BDISOF09), respectively)**.** * represents significance at 5% level of significance. Similar letters at the top of bars indicate the treatment means are statistically similar.

*OsPR1* showed maximum (2.6) levels of relative expression when plants were treated with BDISOF91 (*T. erinaceum*), followed by BDISOF67 (*T. paraviridescens*), BDISOF08 (*T. asperellum*), and BDISOF09 (*T. asperellum*) at 144, 72, 24, and 24 HAI, respectively, as compared to control (Figure 5A). The highest (6.63) expression of *OsPR10* was recorded in BDISOF91 (*T. erinaceum*), followed by BDISOF08 (*T. asperellum*), BDISOF09 (*T. asperellum*), and BDISOF67 (*T. paraviridescens*) at 48, 48, 24, and 24 HAI, respectively, as compared to control (Figure 5B). On the other hand, *OsWRKY45* showed maximum (7.91) levels of relative expression when plants were treated with BDISOF67 (*T. paraviridescens*), BDISOF09 (*T. asperellum*), BDISOF08 (*T. asperellum*), and BDISOF91 (*T. erinaceum*) at 24 HAI for each treatment as compared to control (Figure 5C), while the highest (2.98) expression of *OsWRKY62* was recorded in BDISOF91 (*T. erinaceum*), followed by BDISOF09 (*T. asperellum*), BDISOF08 (*T. asperellum*), and BDISOF67 (*T. paraviridescens*) at 144, 24, 24, and 144 HAI, respectively, as compared to control (Figure 5D). Moreover, *OsWRKY71* showed maximum (6.69) levels of relative expression when plants were treated with BDISOF91 (*T. erinaceum*), followed by BDISOF09 (*T. asperellum*), BDISOF67 (*T. paraviridescens*), and BDISOF08 (*T. asperellum*) at 24 HAI for each treatment as compared to control (Figure 5E). The highest (6.54) expression of *OsHI-LOX* was recorded in BDISOF08 (*T. asperellum*), followed by BDISOF09 (*T. asperellum*), BDISOF91 (*T. erinaceum*), and BDISOF67 (*T. paraviridescens*) at 24 HAI for each treatment as compared to control (Figure 5F). Furthermore, *OsACS2* showed maximum (4.81) levels of relative expression when plants were treated with BDISOF67 (*T. paraviridescens*), followed by BDISOF08 (*T. asperellum*), BDISOF09 (*T. asperellum*), and BDISOF91 (*T. erinaceum*) at 48 HAI for each treatment as compared to control (Figure 5G). These results could somewhat indicate the systemic protection of rice plants against *X. oryzae* pv*. oryzae* due to the induction of resistance by different *Trichoderma* strains, and an increase in the above defense-related genes related to the SA and JA pathways.

## 3. Discussion

Rhizosphere microorganisms that are beneficial to plants can greatly enhance their resistance to pathogens by inducing the production of defense genes and proteins [11,15,39,40,57]. *Trichoderma* isolates in plants have been shown to protect plants from biotic stressors by, among other mechanisms, inducing systemic resistance, programmed cell death, triggering signaling cascades, callose deposition, induction of phytoalexins and other secondary metabolites, and producing antibacterial reactive oxygen species [13,58,59,60]. The findings of this study show that when infected with *X. oryzae* pv. *oryzae*, rice plants primed with beneficial *Trichoderma* strains had higher activity of defense-related enzymes and genes related to SA and JA pathways in leaves compared to control plants.

In the current study, the lesion length of rice leaves was reduced, ranging from 31.25% (BDISOF09: *T. asperellum)* to 87.5% (BDISOF08: *T. asperellum*) in plants treated with *Trichoderma* isolates. This significant reduction in lesion development in the *in vitro* pathogenicity assay signifies that the rice plants primed with *Trichoderma* successfully protected against the development of bacterial blight disease. The findings of this study correlate with the reports from Jambhulkar et al., Gangwar (2013), and Manmeet and Thind, who reported that *Trichoderma* species were found to reduce the lesion length as well as the severity of bacterial blight diseases in rice plants [18,19,61]. However, Divya et al. revealed that a *T. asperellum* isolate was 17.20% more effective in controlling lesion length development than *T. atroviride* [57]. Meanwhile, *T. harzianum* was found to be the best in managing bacterial blight, giving 52.66% and 26.66% reduction in disease severity and incidence, respectively [62]. In another study, Kariuki et al. found that *Trichoderma* isolate T1 was found to be the most effective in reducing bacterial wilt incidence by more than 61.66% compared to the control [63]. Interestingly, *T. paraviridescens* (BDISOF67) and *T. erinaceum* (BDISOF91) were able to significantly reduce the radial growth of *X. oryzae* pv*. oryzae* before coming in direct contact with *Trichoderma* strains. This might be because of the fact that *Trichoderma* strains release antimicrobial compounds in the medium, which in turn reduces or stops the multiplication rate of *X. oryzae* pv*. oryzae* in vitro [11,64,65,66]. A similar report by Gangwar and Sinha demonstrated that *Trichoderma* spp. was able to inhibit the radial growth of *X. oryzae* pv. *oryzae* at a maximum of 100% [13]. As stated by Kannan et al., among different isolates of *Trichoderma*, TAIK 1 was found to significantly inhibit more than 40% growth of *X. oryzae* pv*. oryzae in vitro* [8]. A similar trend was observed by Rubio et al., who revealed that *T. parareesei* showed antagonistic action against *Pythium irregulare* due to its production of cellulolytic enzymes, which break down the cellulose-based cell walls of the fungus [67].

A key discovery of this research is the mechanism by which *Trichoderma* strains reduce the bacterial blight of rice by inducing resistance against *X. oryzae* pv. *oryzae*. We noticed an enhanced activity of defense-related enzymes, viz. PAL, CAT, PPO, and POD, in rice on seed priming with *Trichoderma* strains. Upregulation of these enzymes leads to the production of signaling molecules, like SA and JA, and metabolites with defense functions, including phenols, phytoalexins, lignin, as well as flavonoids, and antimicrobial activities in plants [34,59,68,69]. Our results show PAL activity was upregulated a maximum of four times over control at 72 HAI when plants were treated with *T. erinaceum* and *T. asperellum*. These findings, on the whole, are in agreement with the findings of [39,70,71,72]. Enhanced activity of defense-related enzymes was also reported by Adss et al., who showed that POD, PPO, and PAL activity were significantly increased over control in all treatments after treating *A. solani* with *T. harzianum* [37]. Similar outcomes were observed while inducing the activity of PPO and POD in chickpeas [22] and rice [40] treated with *T. harzianum* and *Bacillus* spp., respectively, against *Sclerotium rolfsii* and *Pyricularia oryzae* infection. Mei et al. discovered that cucumbers treated with *Trichoderma* strains against *Fusarium* wilt had a higher CAT expression level [73]. In contrast, plants inoculated with *T. asperellum* for this study expressed elevated levels of catalase enzyme in leaves at 24 HAI over control, which coincides with Samal et al., who reported that biopriming with *T. erinaceum* resulted in reduced bacterial blight by inducing CAT activity in rice against *X. oryzae* pv. *oryzae* [38]. These findings clearly support that PAL, CAT, PPO, and POD activity might be significant enzymes for the induction of disease resistance to *X. oryzae* pv*. oryzae* in rice plants. The next step of our research is to investigate the expression of other defense-related enzymes in rice plants treated with *Trichoderma* strains.

RT-PCR was employed in this study to evaluate the expression patterns of several defense-related genes (*OsPR1*, *OsPR10*, *OsWRKY45*, *OsWRKY62*, *OsWRKY71*, *OsHI-LOX*, and *OsACS2*) that were reported to be induced by niclosamide, a chemical inducer in rice plants treated with different *Trichoderma* strains [41]. Our results clearly indicate that the expression of the genes, viz., *OsWRKY45*, *OsWRKY62*, *OsWRKY71*, *OsHI-LOX*, and *OsACS2*, were significantly higher at 144 HAI after *X. oryzae* pv. *oryzae* infection, in contrast to other time course expressions, suggesting that they might be responsible for the enhanced resistance to bacterial blight in rice plants. No major changes in *OsPR1* and *OsPR10* expression were observed in plants treated with different *Trichoderma* strains, indicating that these genes might not be primarily responsible for the enhanced resistance of rice plants to bacterial blight. These results are consistent with those reported by [41,46,49,67,74]. Meanwhile, Contreras-Cornejo et al. revealed that the expression of *Lox2 A* genes in response to *T. virens* or *T. atroviride* indicated the involvement of SA and/or JA pathways in the defense signaling pathway activated by those fungi [49]. However, our analysis revealed an antagonistic relationship between the genes of the SA and JA synthesis pathways, in which the presence of *OsHI-LOX* and *OsACS2* in the JA assay suppressed the expression of *OsPR1* and *OsPR10* in the SA assay. This strategy is backed up by Mur et al., who reported that SA and JA act antagonistically when used in higher concentrations [75]. In contrast, De Vleesschauwer et al. developed a model that shows how SA and JA pathways might combine to form a shared defense mechanism that is effective against various sorts of invaders [76].

Confirmation through the endpoint of qRT-PCR complements the expression of defense-related genes involved in SA and JA acid pathways as the maximum expression after 24 h of inoculation with all tested *Trichoderma* strains. This strategy is also supported by Ding et al., who showed that *PR-1.1* was induced in response to treatments with SA or methyl jasmonate *(MeJA),* while *PR3* and *LOX2* responded positively to MeJA treatment after 24 h [47]. In addition, overexpression of LOX genes has been demonstrated to be necessary for giving resistance to diseases caused by bacteria [44,45,77]. In another study, Uji et al. stated that the *Trichoderma* strain induced the expression of Lox1 genes against the rice bacterial blight pathogen *X. oryzae* pv. *oryzae* [78]. In our study, higher levels of expression of the *OsWRKY45, OsWRKY62,* and *OsWRKY71* genes were observed in plants raised from treated seeds and sprayed with different *Trichoderma* strains. These findings are totally in agreement with Kim et al., who demonstrated that niclosamide blocks the development of rice leaf blight by inducing the expression of defense-related genes, including *OsPR1*, *OsPR10*, *OsWRKY45*, *OsWRKY62*, *OsWRKY71*, *OsHI-LOX,* and *OsACS2* [38]. However, some studies show that multiple members of the WRKY gene family involved in JA response pathways, such as WRKY, bZIP [20], OsWRKY80, OsWRKY4 [48], OsWRKY9, OsWRKY45, OsWRKY5, OsWRKY28, OsWRKY29 [79], WRKY18, WRKY40 [80], and OsWRKY45 [81] activated the defense response in the interaction with the beneficial fungus *Trichoderma* species on susceptible rice plants. Together with the findings, we support that rice plants treated with beneficial *Trichoderma* species can significantly induce resistance against *X. oryzae* pv. *oryzae* by the up-regulation of defense-related enzymes and marker genes of the SA and JA pathways. Conversely, it would be interesting for our study to test the SA and JA levels in *Trichoderma*-treated rice plants.

## 4. Materials and Methods

### 4.1. Identification of Trichoderma Species Antagonistic to X. oryzae pv. oryzae

To identify the *Trichoderma* species antagonistic to *X. oryzae* pv. *oryzae,* rice plant samples with root systems were collected from 40 rice growing districts, representing 30 agro-ecological zones of Bangladesh. The *Trichoderma* species were then isolated following the dilution plate technique (up to 10^−3^ concentration) on PDA (Potato Dextrose Agar; Difco™) plates at a temperature of 28 °C. To assess the antagonistic activity of *Trichoderma* species against *X. oryzae* pv. *oryzae,* in vitro growth assay was performed as described by Tian et al. [82] with slight modifications. Briefly, *X. oryzae* pv. *oryzae* inocula were prepared from 48 h old cultures and a 100 µL bacterial cell suspension (1 × 10^8^ cells/ml) was spread on NBY (Nutrient Broth Yeast) plates. A 5 mm mycelial block of *Trichoderma* species was then placed at the centre of the plate and incubated at 28 °C for 3 days. The growth inhibition of *X. oryzae* pv*. oryzae* by the isolates of *Trichoderma* as indicated by clear halo zones were then recorded and expressed in percentage over control using the following formula:

$$\%\;Growth\;Inhibition=\frac{\mathrm{Diameter}\;\mathrm{of}\;\mathrm{fungal}\;\mathrm{colony}\;\mathrm{with}\;\mathrm{clear}\;\mathrm{halo}\;\mathrm{zone}\;-\;\mathrm{Diameter}\;\mathrm{of}\;\mathrm{the}\;\mathrm{fungal}\;\mathrm{colony}}{\mathrm{Diameter}\;\mathrm{of}\;\mathrm{fungal}\;\mathrm{colony}\;\mathrm{with}\;\mathrm{clear}\;\mathrm{halo}\;\mathrm{zone}}$$

The genomic DNA of these *Trichoderma* isolates was then extracted by using Wizard^®^ Genomic DNA Purification Kit solution (Promega, Madison, WI, USA) according to the manufacturer’s instructions. PCR reactions were performed with the universal primer sets ITS 1 (5′-TCCGTAGGTGAACCTGCGG-3′) and ITS 4 (5′TCCTCCGCTTATTGATATGC-3′) according to White et al. [83]. The PCR amplification was performed in a T100 thermal cycler (BioRed) using the initial denaturation at 95 °C for 5 min, denaturation at 95 °C for 30 s, annealing at 55 °C for 1 min, extension at 72 °C for 1 min for 35 cycles and final extension at 72 °C for 6 min. The molecular size of the resulting PCR product was analyzed on a 1.0% agarose gel. PCR-amplified internal transcribed spacer (ITS) regions were then subjected to partial nucleotide sequencing using forward primer ITS1 in the Macrogen Lab, South Korea, via Biotech Concern Bangladesh. The DNA sequences were then compared using the BLAST (Basic Local Alignment Search Tool) program to identify their closest relatives.

### 4.2. Formulation of Trichoderma Species, Seed Priming, and Foliar Spray of Formulated Trichoderma Species

A mycelial disc (5 mm diameter) of each *Trichoderma* isolate was inoculated in 100 mL PDB broth. The number of colony-forming units was counted after 7 days. The mycelial mat along with conidia from potato dextrose broth (PDB) was mixed thoroughly with autoclaved talcum powder pretreated with 0.5% carboxy methyl cellulose (CMC) (5 g CMC dissolved in 100 mL water mixed with 1 kg talcum powder). The mixture was air-dried in a laminar flow hood and kept in plastic bags at 30% moisture content. The formulated fungal antagonists were then kept at 4–8 °C in the refrigerator. The rice seeds of IR24 were then treated with *Trichoderma* formulations at 10 g per kg rice seeds for half an hour. Treated seeds were sown in the earthen pots, and then 30 days old seedlings were transplanted in the pot following a completely randomized design (CRD) with 3 replications. The *Trichoderma* formulations were then sprayed at 40, 50, 65, and 75 days after transplanting (DAT). Formulated fungal powders were suspended in water to prepare the fungal solution at 0.5%, i.e., 5 g/L water and sprayed on the plant surface with the help of a sprayer. 

### 4.3. Inoculation of Rice Plants with X. oryzae pv. oryzae

The *X. oryzae* pv*. oryzae* was cultured in NBY agar medium at 28 °C for 48 h and then resuspended in sterile distilled water at a cell density of 5 × 10^8^ cells/ml measured by Spectrophotometer. The leaves of each hill in each replication were inoculated by the clip-inoculation method of Kauffman [84]. Artificial inoculation of *X. oryzae* pv. *oryzae* was done at 60 DAT. After inoculating, the pots were transferred to an artificial growth chamber maintaining temperature (28 °C) and relative humidity (90%). Data was collected on lesion length at 7, 14, and 21 days after inoculation (DAI). The best or potential *Trichoderma* antagonistic to *X. oryzae* pv*. oryzae* was identified by the data analysis.

### 4.4. Expression Analysis of Some Defense-Related Enzymes in Rice in Response to X. oryzae pv. oryzae

Ten rice leaf samples for each treatment were collected at 24, 48, 72, and 144 h after inoculation (HAI) in zipper bags. The collected leaf samples were frozen with liquid nitrogen and ground into powder for either RNA extraction immediately, or the ground samples were stored at −80 °C for future use. Then, to explore the mechanism of *Trichoderma* mediated induced resistance in rice against *X. oryzae* pv*. oryzae,* the activities of core defense-related enzymes, viz., PAL, CAT, PPO, and POD, were assessed. 

#### 4.4.1. Phenylalanine Ammonia-Lyase (PAL) 

The PAL activity was analyzed at 290 nm wavelength by measuring the conversion of L-phenylalanine into ammonia and trans-cinnamic acid [85]. For this, 1 g rice leaves was homogenized in 5 mL of 0.1 M borate buffer, pH 7.0, containing 0.1 g polyvinyl polypyrrolindone (PVP) at 4 °C with a pestle and mortar. The homogenate was centrifuged at 10,000× *g* for 30 min at 4 °C. The supernatant was collected and used in the enzyme assay. The reaction mixture included 0.4 mL of enzyme extract, 0.5 mL of 0.1 M borate buffer (pH 8.8), and 0.5 mL of 12 nm L-phenylalanine in the same buffer. The reaction mixture was incubated at 30 °C for 30 min in a water bath. The reference cell was 0.4 mL of enzyme extract and 1.0 mL of borate buffer. The amount of trans-cinnamic acid synthesized was calculated using its absorption coefficient of 9630 µmol s^−1^ g^−1^. 

#### 4.4.2. Catalase (CAT) 

To study the CAT activity, the decreased amount of hydrogen peroxide (H_2_O_2_) was determined at 240 nm wavelength [86]. “The CAT assay mixture of 3 mL consisted of 0.05 mL extract, 1.5 mL phosphate buffer (100 mM buffer, pH 7.0), 0.5 mL H_2_O_2_, and 0.95 ml distilled water. A decrease in the absorbance was recorded at 240 nm. The CAT activity was expressed as μmol of H_2_O_2_ oxidized per minute per gram FW”.

#### 4.4.3. Polyphenol Oxidase (PPO) 

The PPO was determined at 280 nm wavelength using L-tyrosine as the substrate [87]. For this, 0.5 mL of leaf extracts were used for the PPO (catechol oxidase) assay. It was mixed with 250 μL of 50 mM sodium phosphate buffer. The rate of increase in absorbance at 525 nm was measured for 2 min after the addition of 500 μL 0.1 M catechol. The OD_525_ was measured using a T80 UV/VIS Spectrophotometer (pg instruments). There were 3 replications for each sample. One unit of the enzyme was defined as the amount of enzyme that increases the OD_525_ value by 0.01. 

#### 4.4.4. Peroxidase (POD)

The POD was assayed using guaiacol as a substrate at 470 nm wavelength and expressed as changes in absorbance min^−1^ g^−1^ fresh wt of tissue [88]. Rice leaves (1 g) were homogenized in 5 mL 0.1M phosphate buffer (pH 7.0) at 4 °C. Then it was centrifuged at 10,000× *g* for 20 min at 4 °C and supernatant was collected. In a sample cuvette of the spectrophotometer, 1.5 mL of 0.05M pyrogallol and 0.1 mL of enzyme extract were added. The absorbance reading will be adjusted to zero at 420 nm in the T80 UV/VIS Spectrophotometer (pg instruments). To initiate the reaction, 0.1 mL of 1% H_2_O_2_ was added to the sample cuvette. The changes in absorbance were recorded at 30 s intervals.

### 4.5. Assessment of Differential Expression in Plants Treated with Trichoderma 

To study the Salicylic acid (SA) and Jasmonic acid (JA) pathway-mediated induced resistance in rice by *Trichoderma,* a susceptible check variety (IR24) was used. Seven genes were selected for their induced expression by *Trichoderma* which were shown to be induced resistance in rice against BB.

### 4.6. RNA Extraction and cDNA Synthesis

Total RNA was extracted from 30–50 mg of ground rice leaf tissues using the kit SB total RNA isolation system (Promega, Madison, WI, USA) according to the manufacturer’s instructions. First-strand complementary DNA was then synthesized using 5 μg of total RNA using Go-script reverse transcriptase (Promega, Madison, WI, USA) and primers [Oligo(dT) 15 (0.5 µg/reaction) and/or Random Primer (0.5 µg/reaction) or gene-specific primer (10–20 pmol/reaction)]. The following procedures were carried out: primer annealing at 25 °C for 5 min, DNA polymerization at 42 °C for up to 1 h, and deactivation of reverse transcriptase at 70 °C for 15 min. 

### 4.7. Primer and Reverse Transcription (RT)-PCR

RT-PCR was performed using Hot-start Go Taq master mix (Promega, Madison, WI, USA) following the instruction of the kit’s manual. The following primers (Table 7) were used for the analyses of the expression of some selected marker genes of SA and JA pathways.

The PCR amplification reaction was performed under the following thermal cycling conditions: 95 °C for 10 min, 45 cycles of 95 °C for 10 s, 60 °C for 10 s, and 72 °C for 10 s. The expression level of different defense-related genes was compared based on the intensity of the band as compared with the untreated control. The 18S rRNA gene was used as the internal control. The following primers were used (Table 7). 

### 4.8. Real-Time qPCR Assay

The q-PCR assay was performed using Luna q-PCR universal master mix (New England Biolab, London, UK). PCR reaction mix contained Luna universal q-PCR (4.5 µL), forward primer 0.25 µL (10 µm), reverse primer 0.25 µL (10 µm), template cDNA 1 µL, and nuclease-free water 4.0 µL were used for quantification of relative expression. The PCR conditions were as follows: initial denaturation was done at 95 °C for 60 s in one cycle. The denaturation was done at 95 °C for 15 s, extension at 60 °C for 30 s in 40–45 cycles, annealing at 60 °C for 10 s, and melt curve for 60–95 °C for various seconds in one cycle. The relative expression was calculated using the following formula: $\mathrm{Relative}\;\mathrm{expression}\;\mathrm{value}=2^{-\Delta \Delta CT}$ [89]. CT value of the target gene was normalized using the CT value of 18S rDNA. 

### 4.9. Statistical Analysis

The data were subjected to analysis of variance, and Duncan’s multiple range test was used to differentiate means at 5%. The error bars in all figures indicate the standard error of the mean. For data analysis, Minitab (version 17) software was used.

## 5. Conclusions

**Figure 6:**
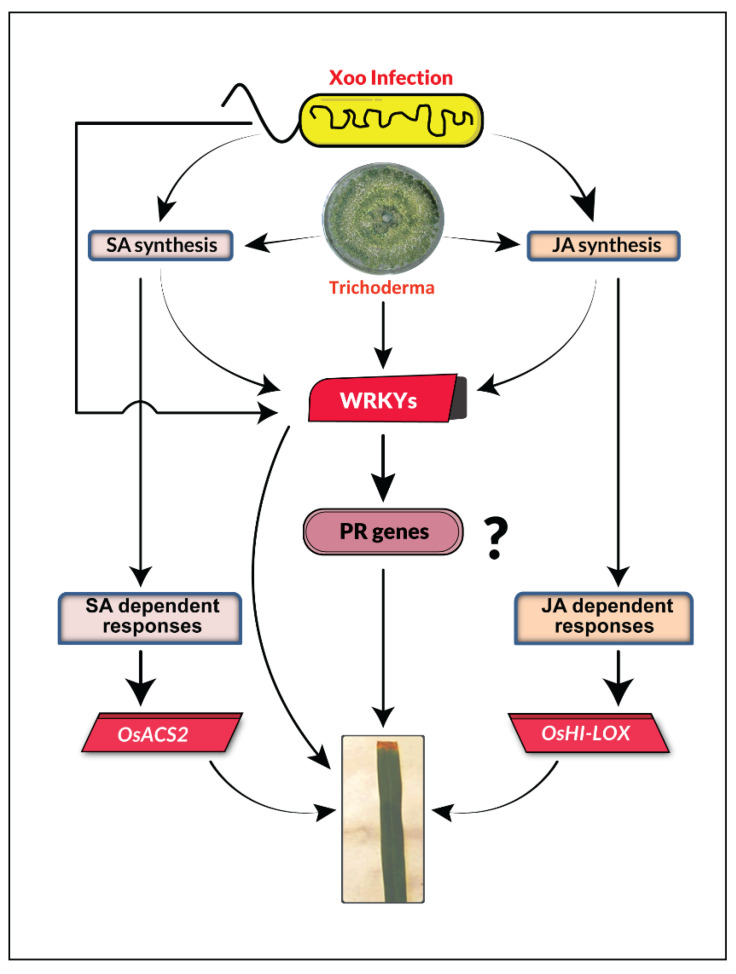
A proposed model illustrating *Trichoderma* mediated induced resistance in rice against *X. oryzae* pv. *oryzae* through triggering the SA and JA pathway related genes and WRKY factors.

Our study offers a glimpse into the molecular mechanism of *Xoo*-rice interactions with *Trichoderma* isolates, and reveals the upregulated expression of the selected defense related enzymes and genes related to the SA and JA pathways. Based on the key findings, *Trichoderma* mediated induced resistance in rice against *Xoo* was summarized using the following proposed model (Figure 6). 

In this proposed model (Figure 6), *Trichoderma* triggers the SA and JA pathways and enhances the expression of some SA and JA responsive genes, and thus induced resistance in rice against *Xoo*. All four *Trichoderma* strains used in this study also enhance the expression of WRKY factors which might be either SA/JA-dependent or independent (Figure 6). However, expression levels of *PR1* and *PR10* genes primarily indicated a weak antagonism of SA and JA pathways in rice against *Xoo* (Figure 6). Therefore, an RNA-seq study is required to understand the underlying mechanisms by which these *Trichoderma* strains promote induced system resistance (Figure 6).

## Figures and Tables

**Table 1:** Closest relatives of the beneficial fungi obtained from rhizosphere samples collected in Boro season 2019 using sequences of the ITS region by Blast program.

Isolate ID	Accession No.	Closest Relatives	Accession No.	Alignment	Homology
BDFISO67R	OP456159	*T. paraviridescens* strain 36114DRJ	MF782827.1	602/605	99
		*T. erinaceum strain CIB T72*	EU280106.1	602/605	99
BDFISO91R		*T. erinaceum strain QT22079*	KY225644.1	605/610	99
	OP456160	* T. erinaceum * strain QT22077	KY225643.1	605/612	99
		* T. erinaceum * strain CIB T72	EU280106.1	602/605	99
BDISOF08R	OP456157	*T. asperellum* bio-material USM	KU878976.1	298/318	94
BDISOF09R	OP456158	*T. asperellum* isolate 20B	MZ044276.1	359/409	88

**Table 2:** Effect of some selected antagonistic *Trichoderma* isolates on the reduction of lesion length in IR24 (a susceptible check variety) caused by *X. oryzae* pv. *oryzae*.

Treatments	Lesion Length (mm)	Reduction of Lesion Length (%)
Untreated control	23.67a	0.00
Seed priming	6.33d	75.00
*T. paraviridescens*	5.50de	75.00
*T. erinaceum*	10.83c	60.42
*T. asperellum*	3.83e	87.50
*T. asperellum*	15.50b	31.25
Level of significance	*	-
CV (%)	10.31	-

* Represents significance at 5% level of significance. Means were compared by Duncan’s Multiple Range Test (DMRT). Values with the same letters are statistically similar. Lesion lengths were measured at 14 DAI. Data are averages of three replications. Untreated control: seed treatment with only distilled water.

**Table 3:** Effect of selected *Trichoderma* isolates on the induction of Phenylalanine ammonia-lyase (PAL) activity when applied as seed treatment and foliar spray.

Treatment	PAL Activities (µmol s^−1^ g^−1^ *)				Times Increase over Control			
	Hours after Inoculation (HAI)							
	24	48	72	144	24	48	72	144
Untreated control	247 ± 26	239 ± 2	159 ± 3	269.5 ± 5				
Seed priming	257 ± 8	286 ± 5	353 ± 25	313.5 ± 5	1.04	1.2	2.22	1.16
*T. paraviridescens*	600 ± 309	609 ± 368	527 ± 281	599 ± 362	2.42	2.55	3.3	2.22
*T. erinaceum*	587 ± 225	555 ± 199	647 ± 352	605 ± 269	2.37	2.32	4.05	2.25
*T. asperellum*	706 ± 345	622 ± 384	530 ± 288	546 ± 375	2.85	2.6	3.32	2.03
*T. asperellum*	556 ± 249	611 ± 300	682 ± 289	608 ± 314	2.25	2.56	4.27	2.26
Level of significance	NS	NS	NS	NS				
CV (%)	173	173	173	173				

* µmol trans-cinamic acid s^−1^ g^−1^ protein; means were compared by Duncan’s Multiple Range Test (DMRT). Data are averages of three replications. Untreated control: seed treatment with only distilled water.

**Table 4:** Effect of selected *Trichoderma* isolates on the induction of Catalase (CAT) activity when applied as seed treatment and foliar spray.

Treatment	CAT Activities (µmol H_2_O_2_ min^−1^ g^−1^)				Times Increase over Control			
	Hours after Inoculation (HAI)							
	24	48	72	144	24	48	72	144
Untreated control	50 ± 5b	150 ± 5bc	121 ± 5a	104 ± 5a				
Seed priming	135 ± 5ab	247 ± 5a	128 ± 5a	53 ± 62b	2.7	1.65	1.06	0.51
*T. paraviridescens*	273 ± 595a	174 ± 2bc	162 ± 46a	218 ± 49b	5.46	1.16	1.34	2.1
*T. erinaceum*	264 ± 88ab	223 ± 45b	118 ± 4a	158 ± 16b	5.28	1.49	0.98	1.52
*T. asperellum*	164 ± 29ab	170 ± 3b	59 ± 23a	201 ± 26b	3.28	1.13	0.49	1.94
*T. asperellum*	271 ± 90ab	113 ± 8c	78 ± 14a	367 ± 19b	5.42	0.75	0.64	3.53
CV (%)	48.75	12.35	18.22	29.71				
Level of Significance	*	*	NS	*				

* Represents significance at 5% level of significance. Means were compared by Duncan’s Multiple Range Test (DMRT). Values with the same letter are statistically similar. Data are averages of three replications. Untreated control: seed treatment with only distilled water.

**Table 5:** Effect of selected *Trichoderma* isolates on the induction of Polyphenoloxidase (PPO) activity when applied as seed treatment and foliar spray.

Treatment	PPO Activities (Units g^−1^ min^−1^ FW)				Times Increase over Control			
	Hours after Inoculation (HAI)							
	24	48	72	144	24	48	72	144
Untreated control	247 ± 26b	239 ± 2	159 ± 3c	269 ± 5ab				
Seed priming	257 ± 8b	286 ± 5	353 ± 25a	313 ± 5a	1.03	1.2	2.21	1.16
*T. paraviridescens*	454 ± 92a	257 ± 15	247 ± 1b	254 ± 17ab	1.77	1.07	1.55	0.94
*T. erinaceum*	434 ± 71ab	311 ± 45	271 ± 24ab	296 ± 40ab	0.95	1.3	1.7	1.10
*T. asperellum*	428 ± 67ab	276 ± 38	250 ± 7b	222 ± 50b	0.98	1.15	1.58	0.82
*T. asperellum*	403 ± 96ab	284 ± 27	324 ± 69ab	274 ± 20ab	0.94	1.19	2.03	1.01
LSD	61.04	24.41	28.24	25.49				
CV (%)	173.25	173.13	173.48	173.32				
Level of Significance	*	NS	*	*				

* Represents significance at 5% level of significance. Values with the same letters are statistically similar. Data are averages of three replications. Untreated control: seed treatment with only distilled water.

**Table 6:** Effect of selected *Trichoderma* isolates on the induction of Peroxidase (POD) activity when applied as seed treatment and foliar spray.

Treatment	POD Activities (Units min^−1^ g^−1^ FW)				Times Increase over Control			
	Hours after Inoculation (HAI)							
	24	48	72	144	24	48	72	144
Untreated control	760 ± 60b	721 ± 55	1009 ± 29ab	1191 ± 55				
Seed priming	759 ± 55b	851 ± 45	1186 ± 100ab	1224 ± 0	0.99	1.18	1.17	1.03
*T. paraviridescens*	850 ± 99b	1118 ± 28	1503 ± 232ab	1751 ± 1004	1.12	1.55	1.49	1.47
*T. erinaceum*	831 ± 138b	880 ± 281	1375 ± 486ab	1866 ± 794	1.09	1.22	1.36	1.57
*T. asperellum*	847 ± 237b	736 ± 138	697 ± 425b	1809 ± 151	1.11	1.02	0.69	1.52
*T. asperellum*	1239 ± 109.5a	762 ± 288	1704 ± 42a	1338 ± 692	1.63	1.06	1.69	1.12
CV (%)	107.35	162.37	285.25	460.37				
Level of Significance	*	NS	*	NS				

* Represents significance at 5% level of significance. Means were compared by Duncan’s Multiple Range Test (DMRT). Values with the same letters are statistically similar. Data are averages of three replications. Untreated control: seed treatment with only distilled water.

**Table 7:** List of primers for the expression study of some selected defense-related genes of rice against *X*. *oryzae* pv. *oryzae*.

Genes	Primer Name	Sequence (5′-3′)	Marker Gene
OsPR1	OsPR1forward	TTATCCTGCTGCTTGCTGGT	SA pathway
	OsPR1 reverse	GATGTTCTCGCCGTACTTCC	
OsPR10	OsPR10 forward	GGCACCATCTACACCATGAA	SA pathway
	OsPR10 reverse	TTGTCGG CTGTGATGA ATGT	
OsWRKY45	OsWRKY45 forward	CCGGCATGGAGTTCTTCAAG	SA pathway
	OsWRKY45 reverse	TATTT CTGTACACACGCGTGGAA	
OsWRKY62	OsWRKY62 forward	AGGATGGGTACCAATGGA	SA pathway
	OsWR KY62 reverse	ACGAGTTGATGGAGATGGA	
OsWRKY71	OsWRKY71 forward	AGCCCAA GA TCTCC AAGCTC	SA pathway
	OsWRKY71 reverse	ACGAGGATCGTGTTGTCCTC	
OsACS2	OsACS2 forward	GGAATAAAGCTGC TGCCGAT	SA pathway
	OsACS2 reverse	TGAGCCTGAAG TCGTTGAAGC	
OsHI-LOX	OsHI-LOX forward	GCATCCCCAACAGCACATC	JA Pathway
	OsHI-LOX reverse	AATAAAGATTTGGGA GTGACATATTGG	
18S rRNA	18S rRNA Forward	CTACGTCCCTGCCCTTTGTACA	
	18S rRNA Reverse	ACACTTCACCGGACCATTCAA	

## Data Availability

The data is contained within the article and the Supplementary Materials.

## Supplementary Materials

The following supporting information can be downloaded at: https://www.mdpi.com/article/10.3390/plants12091864/s1.

## References

[1] Vaughan D.A., Morishima H., Kadowaki K. (2003). Diversity in the Oryza genus. Curr. Opin. Plant Biol..

[2] Szareski V.J., Carvalho I.R., da Rosa T.C., Dellagostin S.M., de Pelegrin A.J., Barbosa M.H., dos Santos O.P., Muraro D.S., de Souza V.Q., Pedó T. (2018). Oryza Wild Species: An alternative for rice breeding under abiotic stress conditions. Am. J. Plant Sci..

[3] BBS (Bangladesh Bureau of Statistics) (2020). 2020: Yearbook of Agricultural Statistics.

[4] Ansari T.H., Ahmed M., Akter S., Mian M.S., Latif M.A., Tomita M. (2019). Estimation of rice yield loss using a simple linear regression model for bacterial blight disease. Bangladesh Rice J..

[5] Haq M., Mia M.A.T., Rabbi M.F., Ali M.A., Lal R., Sivakumar M.V.K., Faiz M.A., Rahman A.H.M.M., Islam K.R. (2010). Incidence and severity of rice diseases and insect pests in relation to climate change. Climate Change and Food Security in South Asia.

[6] Niño-Liu D.O., Ronald P.C., Bogdanove A.J. (2006). *Xanthomonas oryzae* pathovars: Model pathogens of a model crop. Mol. Plant Pathol..

[7] Mew T.W., Alvarez A.M., Leach J.E., Swings J. (1993). Focus on bacterial blight of rice. Plant Dis..

[8] Chukwu S.C., Rafii M.Y., Ramlee S.I., Ismail S.I., Hasan M.M., Oladosu Y.A., Magaji U.G., Akos I., Olalekan K.K. (2019). Bacterial leaf blight resistance in rice: A review of conventional breeding to molecular approach. Mol. Biol. Rep..

[9] Yasmin S., Hafeez F.Y., Mirza M.S., Rasul M., Arshad H.M., Zubair M., Iqbal M. (2017). Biocontrol of bacterial leaf blight of rice and profiling of secondary metabolites produced by rhizospheric *Pseudomonas aeruginosa* BRp3. Front Microbiol..

[10] Kumari K.A., Kumar K.N.R., Rao C.N. (2014). Adverse effect of chemical fertilizers and pesticides on human health and environment. J. Chem. Pharmaceut. Sci..

[11] Kannan C., Mishra D., Rekha G., Maruthi P., Shaik H., Sundaram R.M. (2021). Diversity analysis of antagonistic microbes against bacterial leaf and fungal sheath blight diseases of rice. Egypt. J. Biol. Pest Control..

[12] Shobha B., Lakshmeesha T.R., Ansari M.A., Almatroudi A., Alzohairy M.A., Basavaraju S., Alurappa R., Niranjana S.R., Chowdappa S. (2020). Mycosynthesis of ZnO nanoparticles using *Trichoderma* spp. isolated from rhizosphere soils and its synergistic antibacterial effect against *Xanthomonas oryzae* pv. *oryzae*. J. Fungi.

[13] Gangwar G.P., Sinha A.P. (2012). Evaluation of *Trichoderma* spp. and fluorescent pseudomonads for the management of bacterial leaf blight of rice. Indian Phytopath..

[14] Mishra D., Rajeswari B., Rao P.R., Maheswari T.U., Kannan C. (2021). Friendly microbes help rice to grow and suppress its pathogens: *Trichoderma* and *Bacillus* Vs *Xanthomonas* in rice. Environ. Conserv. J..

[15] Jain A., Chatterjee A., Das S. (2020). Synergistic consortium of beneficial microorganisms in rice rhizosphere promotes host defense to blight-causing *Xanthomonas oryzae* pv. *oryzae*. Planta.

[16] Abo-Elyousr K.A., Khalil Bagy H.M., Hashem M., Alamri S.A., Mostafa Y.S. (2019). Biological control of the tomato wilt caused by *Clavibacter michiganensis* subsp. *michiganensis* using formulated plant growth-promoting bacteria. Egypt. J. Biol. Pest Control..

[17] Wilson M., Lindow S.E. (1993). Release of recombinant microorganisms. Annu. Rev. Microbial..

[18] Jambhulkar P.P., Sharma P., Manokaran R., Lakshman D.K., Rokadia P., Jambhulkar N. (2018). Assessing synergism of combined applications of *Trichoderma harzianum* and *Pseudomonas fluorescens* to control blast and bacterial leaf blight of rice. Eur. J. Plant Pathol..

[19] Gangwar G.P. (2013). Field efficacy of formulation of fungal bioagents against bacterial leaf blight of rice caused by *Xanthomonas oryzae* pv. *oryzae* (Uyeda and Ishiyama) Dowson. J. Appl. Nat. Sci..

[20] Wu G., Zhang Y., Wang B., Li K., Lou Y., Zhao Y., Liu F. (2021). Proteomic and Transcriptomic Analyses Provide Novel Insights into the Crucial Roles of Host-Induced Carbohydrate Metabolism Enzymes in *Xanthomonas oryzae* pv. *oryzae* Virulence and Rice-Xoo Interaction. Rice.

[21] Ryan E.P., Heuberger A.L., Weir T.L., Barnett B., Broeckling C.D., Prenni J.E. (2011). Rice bran fermented with *Saccharomyces boulardii* generates novel metabolite profiles with bioactivity. J. Agric. Food Chem..

[22] Singh R.R., Chinnasri B., De Smet L., Haeck A., Demeestere K., Van Cutsem P., Van Aubel G., Gheysen G., Kyndt T. (2019). Systemic defense activation by COS-OGA in rice against root-knot nematodes depends on stimulation of the phenylpropanoid pathway. Plant Physiol. Biochem..

[23] Kloepper J.W., Tuzun S., Liu L., Wei G. (1993). Plant growth-promoting rhizobacteria as inducers of systemic disease resistance. Pest Manag. Biol. Based Technologies. Am. Chem. Soc. Books Wash. DC.

[24] Pieterse C.M., Zamioudis C., Berendsen R.L., Weller D.M., Van Wees S.C., Bakker P.A. (2014). Induced systemic resistance by beneficial microbes. Annu. Rev. Phytopathol..

[25] Sticher L., Mauch-Mani B., Métraux A.J. (1997). Systemic acquired resistance. Annu. Rev. Phytopathol..

[26] Kessler A., Halitschke R., Baldwin I.T. (2004). Silencing the jasmonate cascade: Induced plant defenses and insect populations. Science.

[27] Van Loon L.C. (2007). Plant responses to plant growth-promoting rhizobacteria. New Perspectives and Approaches in Plant Growth-Promoting Rhizobacteria Research.

[28] Maksimov I.V., Valeev A.S., Cherepanova E.A., Burkhanova G.F. (2014). Effect of chitooligosaccharides with different degrees of acetylation on the activity of wheat pathogen-inducible anionic peroxidase. Appl. Biochem. Microbiol..

[29] van Loon L.C., Rep M., Pieterse C.M. (2006). Significance of inducible defense-related proteins in infected plants. Annu. Rev. Phytopathol..

[30] Hammond-Kosack K.E., Jones J.D.G. (1996). Resistance gene-dependent plant defense responses. Plant Cell Rep..

[31] Choodamani M.S., Hariprasad P., Sateesh M.K., Umesha S. (2009). Involvement of catalase in bacterial blight disease development of rice caused by Xanthomonas oryzae pv. oryzae. Int. J. Pest Manag..

[32] Sofo A., Scopa A., Nuzzaci M., Vitti A. (2015). Ascorbate peroxidase and catalase activities and their genetic regulation in plants subjected to drought and salinity stresses. Int. J. Mol. Sci..

[33] Sharma S.D., Kumar P., Raj H., Bhardwaj S.K. (2009). Isolation of arbuscular mycorrhizal fungi and *Azotobacter chroococcum* from local litchi orchards and evaluation of their activity in the air-layers system. Sci. Hortic..

[34] Kim D.S., Hwang B.K. (2014). An important role of the pepper phenylalanine ammonia-lyase gene (PAL1) in salicylic acid-dependent signalling of the defence response to microbial pathogens. J. Exp. Bot..

[35] Hemm M.R., Rider S.D., Ogas J., Murry D.J., Chapple C. (2004). Light induces phenylpropanoid metabolism in *Arabidopsis* roots. Plant J..

[36] Tahsili J., Sharifi M., Safaie N., Esmaeilzadeh-Bahabadi S., Behmanesh M. (2014). Induction of lignans and phenolic compounds in cell culture of Linum album by culture filtrate of *Fusarium graminearum*. J. Plant Interact..

[37] Adss I.A., Amer G., Bayoumy S.R., Eid R. (2021). Effect of abscisic acid, salicylic acid, potassium silicate, and *Trichoderma harzianum* as biocontrol agent to induce the tomato resistance against early blight disease caused by *Alternaria solani*. Alex. Sci. Exch. J..

[38] Samal P., Mohapatra P.K., Naik S.K., Mukherjee A.K. (2020). Improved photosystem II and defense enzymes activity in rice (*Oryza sativa*) by biopriming against *Xanthomonas oryzae* pv. *oryzae*. Funct. Plant Biol..

[39] Zhang F., Wang Y., Liu C., Chen F., Ge H., Tian F., Yang T., Ma K., Zhang Y. (2019). *Trichoderma harzianum* mitigates salt stress in cucumber via multiple responses. Ecotoxicol. Environ. Saf..

[40] Rais A., Jabeen Z., Shair F., Hafeez F.Y., Hassan M.N. (2017). *Bacillus* spp., a bio-control agent enhances the activity of antioxidant defense enzymes in rice against *Pyricularia oryzae*. PLoS ONE.

[41] Kim S.I., Song J.T., Jeong J.Y., Seo H.S. (2016). Niclosamide inhibits leaf blight caused by *Xanthomonas oryzae* in rice. Sci. Rep..

[42] Conrath U., Beckers G.J., Flors V., García-Agustín P., Jakab G., Mauch F., Newman M.A., Pieterse C.M., Poinssot B., Pozo M.J. (2006). Priming: Getting ready for battle. Mol. Plant-Microbe Interact..

[43] Im J.H., Choi C., Park S.R., Hwang D.J. (2022). The OsWRKY6 transcriptional cascade functions in basal defense and Xa1-mediated defense of rice against *Xanthomonas oryzae* pv. *oryzae*. Planta.

[44] Moon H., Jeong A.R., Kwon O.K., Park C.J. (2022). Oryza-Specific Orphan Protein Triggers Enhanced Resistance to *Xanthomonas oryzae* pv. *oryzae* in Rice. Front. Plant Sci..

[45] Yang J., Duan G., Li C., Liu L., Han G., Zhang Y., Wang C. (2019). The crosstalks between jasmonic acid and other plant hormone signaling highlight the involvement of jasmonic acid as a core component in plant response to biotic and abiotic stresses. Front. Plant Sci..

[46] Saxena A., Mishra S., Ray S., Raghuwanshi R., Singh H.B. (2020). Differential reprogramming of defense network in *Capsicum annum* L. plants against *colletotrichum truncatum* infection by phyllospheric and rhizospheric trichoderma strains. J. Plant Growth Regul..

[47] Ding L.N., Yang G.X., Yang R.Y., Cao J., Zhou Y. (2016). Investigating interactions of salicylic acid and jasmonic acid signaling pathways in monocots wheat. Physiol. Mol. Plant Pathol..

[48] Peng X., Wang H., Jang J.C., Xiao T., He H., Jiang D., Tang X. (2016). OsWRKY80-OsWRKY4 module as a positive regulatory circuit in rice resistance against *Rhizoctonia solani*. Rice.

[49] Contreras-Cornejo H.A., Macías-Rodríguez L., Beltrán-Peña E., Herrera-Estrella A., López-Bucio J. (2011). *Trichoderma*-induced plant immunity likely involves both hormonal-and camalexin-dependent mechanisms in *Arabidopsis thaliana* and confers resistance against necrotrophic fungi *Botrytis cinerea*. Plant Signal. Behave..

[50] Mitsuhara I., Iwai T., Seo S., Yanagawa Y., Kawahigasi H., Hirose S., Ohkawa Y., Ohashi Y. (2008). Characteristic expression of twelve rice PR1 family genes in response to pathogen infection, wounding, and defense-related signal compounds (121/180). Mol. Genet. Genom..

[51] Hwang S.H., Lee I.A., Yie S.W., Hwang D.J. (2008). Identification of an OsPR10a promoter region responsive to salicylic acid. Planta.

[52] Fukushima S., Mori M., Sugano S., Takatsuji H. (2016). Transcription factor WRKY62 plays a role in pathogen defense and hypoxia-responsive gene expression in rice. Plant Cell Physiol..

[53] Qiu D., Xiao J., Xie W., Cheng H., Li X., Wang S. (2009). Exploring transcriptional signalling mediated by OsWRKY13, a potential regulator of multiple physiological processes in rice. BMC Plant Biol..

[54] Ross C.A., Liu Y., Shen Q.J. (2007). The WRKY gene family in rice (*Oryza sativa*). J. Integr. Plant Biol..

[55] Wu K.L., Guo Z.J., Wang H.H., Li J. (2005). The WRKY family of transcription factors in rice and *Arabidopsis* and their origins. DNA Res..

[56] Zhang Y., Wang L. (2005). The WRKY transcription factor superfamily: Its origin in eukaryotes and expansion in plants. BMC Evol. Biol..

[57] Divya M., Rajeswari B., Raghuveer Rao P., Maheswari U.T., Kannan C. (2021). Biological control of bacterial blight of rice using native isolates of *Trichoderma* and *Bacillus* in rice cultivar Telangana Sona (RNR 15048).

[58] Jain A., Singh A., Singh S., Sarma B.K., Singh H.B. (2015). Biocontrol agents-mediated suppression of oxalic acid induced cell death during Sclerotinia sclerotiorum–pea interaction. J. Basic Microbiol..

[59] Yang S.M., Shim G.Y., Kim B.G., Ahn J.H. (2015). Biological synthesis of coumarins in *Escherichia coli*. Microb. Cell Factories.

[60] Shoresh M., Harman G.E., Mastouri F. (2010). Induced systemic resistance and plant responses to fungal biocontrol agents. Annu Rev. Phytopathol..

[61] Manav M., Thind B.S. (2002). Management of bacterial blight of rice with bioagents. Plant Dis. Res.-Ludhiana.

[62] Khan A.A., Rajbir S. (2015). Influence of zinc on *Trichoderma harzianum* and sheath blight of rice under glasshouse conditions. Int. J. Plant Protect..

[63] Kariuki C.K., Mutitu E.W., Muiru W.M. (2020). Effect of *Bacillus* and *Trichoderma* species in the management of the bacterial wilt of tomato (*Lycopersicum esculentum*) in the field. Egypt. J. Biol. Pest Control..

[64] Angraini E., Angraeni D.N., Umami S.S., Sumiati E., Taufiqurokhman T. (2019). Antagonism of *Lentinus Cladopus* Lc4 Extract, *Trichoderma* sp. Jpa Extract on *Bacillus* sp., *Xanthomonas* sp. and *E. coli*. Int. J. Phys. Conf. Ser..

[65] Reino J.L., Guerrero R.F., Hernández-Galán R., Collado I.G. (2008). Secondary metabolites from species of the biocontrol agent *Trichoderma*. Phytochem. Rev..

[66] Verma M., Brar S.K., Tyagi R.D., Surampalli R.N., Valero J. (2007). Antagonistic fungi, *Trichoderma* spp.: Panoply of biological control. Biochem. Eng. J..

[67] Rubio M.B., Quijada N.M., Pérez E., Domínguez S., Monte E., Hermosa R. (2014). Identifying beneficial qualities of *Trichoderma parareesei* for plants. Appl. Environ. Microbiol..

[68] Shoresh M., Yedidia I., Chet I. (2005). Involvement of jasmonic acid/ethylene signaling pathway in the systemic resistance induced in cucumber by *Trichoderma asperellum* T203. Phytopathology.

[69] Chaman M.E., Copaja S.V., Argandoña V.H. (2003). Relationships between salicylic acid content, phenylalanine ammonia-lyase (PAL) activity, and resistance of barley to aphid infestation. J. Agric. Food Chem..

[70] Prathuangwong S., Buensanteai N. (2007). *Bacillus amyloliquefaciens* induced systemic resistance against bacterial pustule pathogen with increased phenols, phenylalanine ammonia lyase, peroxidases and 1, 3-β-glucanases in soybean plants. Acta Phytopathol. Entomol. Hung..

[71] Li S.B., Fang M., Zhou R.C., Huang J. (2012). Characterization and evaluation of the endophyte Bacillus B014 as a potential biocontrol agent for the control of *Xanthomonas axonopodis* pv. *dieffenbachiae*–Induced blight of Anthurium. Biol. Control..

[72] Li Y., Gu Y., Li J., Xu M., Wei Q., Wang Y. (2015). Biocontrol agent *Bacillus amyloliquefaciens* LJ02 induces systemic resistance against cucurbits powdery mildew. Front. Microbiol..

[73] Mei L.I., Hua L.I., Su X.L., Ying T.I., Huang W.K., Jie M.E., Jiang X.L. (2019). The effects of *Trichoderma* on preventing cucumber *fusarium* wilt and regulating cucumber physiology. J. Integr. Agric..

[74] Peng X., Hu Y., Tang X., Zhou P., Deng X., Wang H., Guo Z. (2012). Constitutive expression of rice WRKY30 gene increases the endogenous jasmonic acid accumulation, PR gene expression and resistance to fungal pathogens in rice. Planta.

[75] Mur L.A., Kenton P., Atzorn R., Miersch O., Wasternack C. (2006). The outcomes of concentration-specific interactions between salicylate and jasmonate signaling include synergy, antagonism, and oxidative stress leading to cell death. Plant Physiol..

[76] De Vleesschauwer D., Xu J., Höfte M. (2014). Making sense of hormone-mediated defense networking: From rice to *Arabidopsis*. Front. Plant Sci..

[77] Vidhyasekaran P. (2020). Bioengineering and Molecular Manipulation of Jasmonate Signaling System to Activate Plant Immune System for Crop Disease Management. Plant Innate Immunity Signals and Signaling Systems.

[78] Uji Y., Taniguchi S., Tamaoki D., Shishido H., Akimitsu K., Gomi K. (2016). Overexpression of OsMYC2 results in the up-regulation of early JA-rresponsive genes and bacterial blight resistance in rice. Plant Cell Physiol..

[79] Satoh K., Kondoh H., De Leon T.B., Macalalad R.J., Cabunagan R.C., Cabauatan P.Q., Mauleon R., Kikuchi S., Choi I.R. (2013). Gene expression responses to Rice tungro spherical virus in susceptible and resistant near-isogenic rice plants. Virus Res..

[80] Brotman Y., Landau U., Cuadros-Inostroza A., Takayuki T., Fernie A.R., Chet I., Viterbo A., Willmitzer L. (2013). *Trichoderma*-plant root colonization: Escaping early plant defense responses and activation of the antioxidant machinery for saline stress tolerance. PLoS Pathog..

[81] Qiu Y., Yu D. (2009). Over-expression of the stress-induced OsWRKY45 enhances disease resistance and drought tolerance in *Arabidopsis*. Environ. Exp. Bot..

[82] Tian X.L., Cao L.X., Tan H.M., Zeng Q.G., Jia Y.Y., Han W.Q., Zhou S.N. (2004). Study on the communities of endophytic fungi and endophytic actinomycetes from rice and their antipathogenic activities in vitro. World J. Microbiol. Biotechnol..

[83] White T.J., Bruns T., Lee S.J.W.T., Taylor J. (1990). Amplification and direct sequencing of fungal ribosomal RNA genes for phylogenetics. PCR Protoc. A Guide Methods Appl..

[84] Kauffman H.E. (1973). An improved technique for evaluating resistance of rice varieties to *Xanthomonas oryzae*. Plant Dis. Rep..

[85] Dickerson D.P., Pascholati S.F., Hagerman A.E., Butler L.G., Nicholson R.L. (1984). Phenylalanine ammonia-lyase and hydroxycinnamate: CoA ligase in maize mesocotyls inoculated with Helminthosporium maydis or Helminthosporium carbonum. Physiol. Plant Pathol..

[86] Aebi H. (1984). Catalase in vitro. Methods in Enzymology.

[87] Zhu G.L., Zhong H.W., Zhang A.Q. (1990). Plant Physiological Experimentation.

[88] Hammerschmidt R., Nuckles E.M., Kuć J. (1982). Association of enhanced peroxidase activity with induced systemic resistance of cucumber to *Colletotrichum lagenarium*. Physiol. Plant Pathol..

[89] Livak K.J., Schmittgen T.D. (2001). Analysis of relative gene expression data using real-time quantitative PCR and the 2−ΔΔCT method. Methods.

