# Supplementary material for: Native Trichoderma Induced the Defense-Related Enzymes and Genes in Rice against Xanthomonas oryzae pv. oryzae (Xoo)
Source: Plants (Basel). 2023 Apr 30;12(9):1864. doi: 10.3390/plants12091864 (PMC10180545; doi:10.3390/plants12091864)
Supplement: Supplementary file 1 [file plants-12-01864-s001.zip › plants-2216305-supplementary.pdf]

Supplementary Materials

24HAI

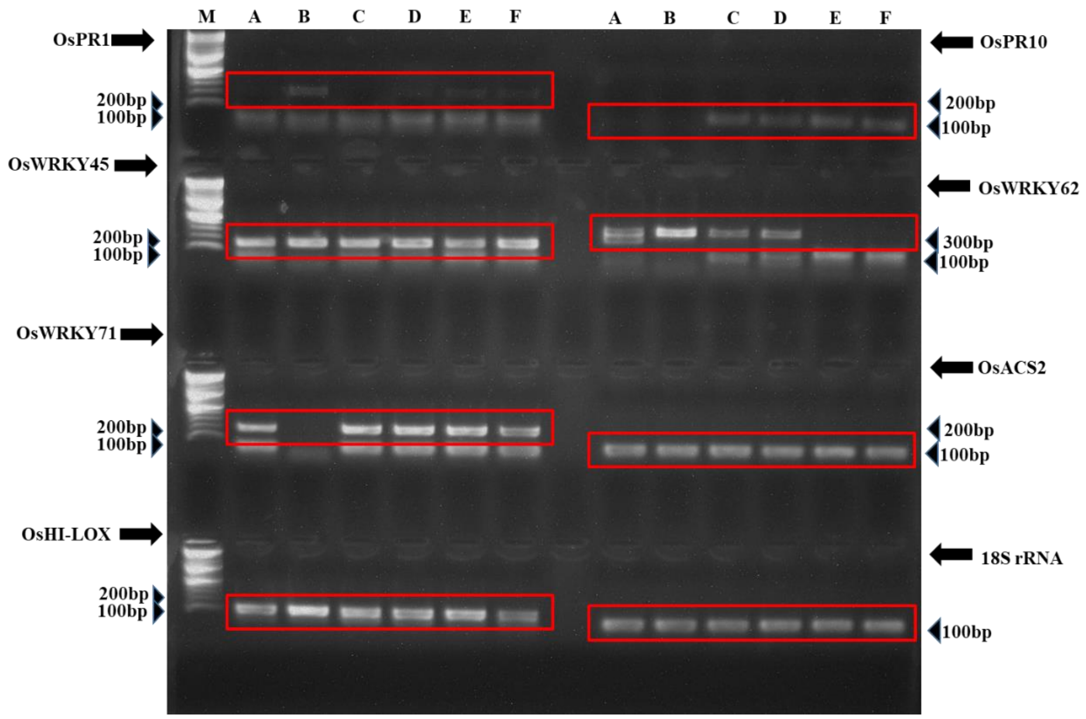

Figure S1. Expression data of some selected marker genes including *OsPR1*, *OsWRKY45*, *OsWRKY71* and *OsHI-LOX* involved in SA and JA acid pathway by RT-PCR at 24 HAI.

48HAI

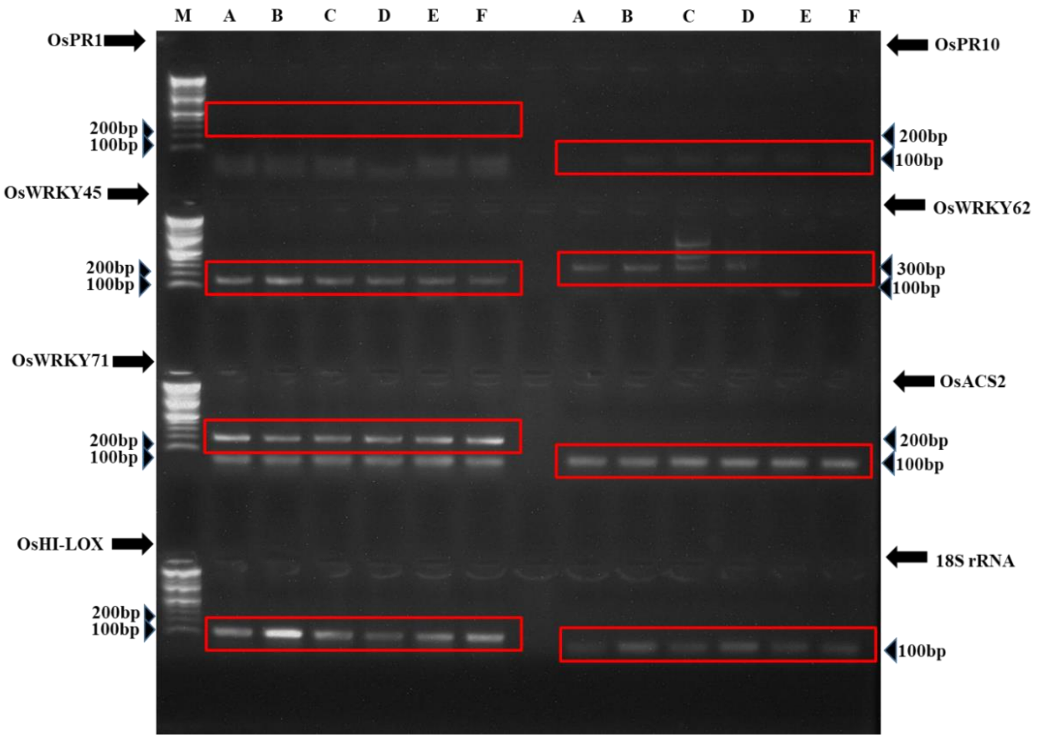

Figure S2. Expression data of some selected marker genes including *OsPR1*, *OsWRKY45*, *OsWRKY71* and *OsHI-LOX* involved in SA and JA acid pathway by RT-PCR at 48 HAI.

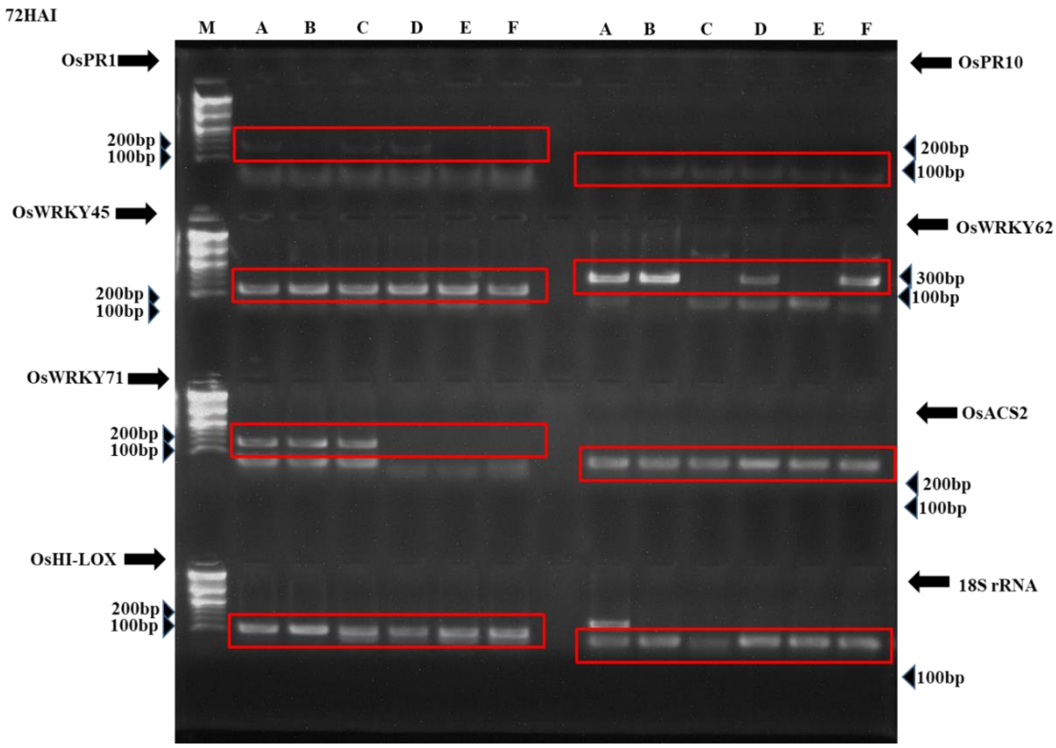

Figure S3. Expression data of some selected marker genes including *OsPR1*, *OsWRKY45*, *OsWRKY71* and *OsHI-LOX* involved in SA and JA acid pathway by RT-PCR at 72 HAI.

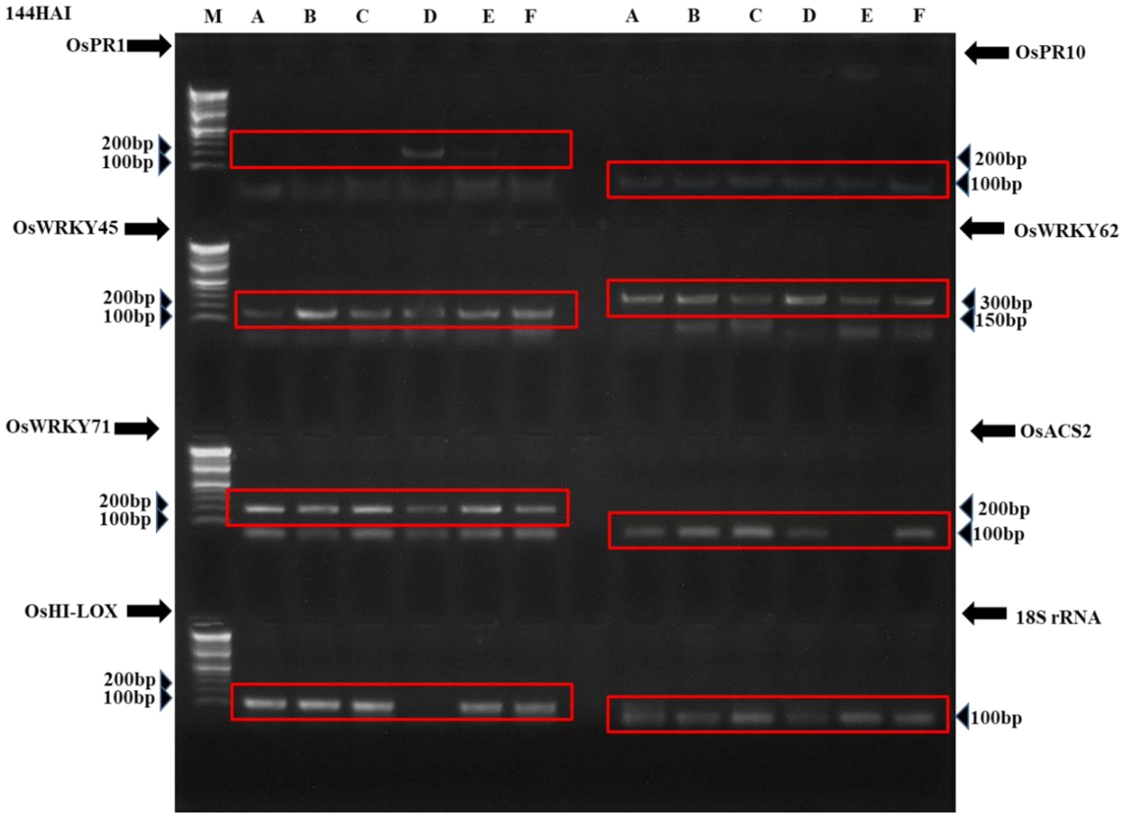

Figure S4. Expression data of some selected marker genes including *OsPR1*, *OsWRKY45*, *OsWRKY71* and *OsHI-LOX* involved in SA and JA acid pathway by RT-PCR at 144 HAI
